# Adnexal Torsion After Isolated Salpingeal Torsion, an Undesired Complication of Conservative Management: A Case Report 

**Published:** 2020-03

**Authors:** Eniola R. Ibirogba, Faheema Abduljalil Alshehabi, Afeefa Ashfaq Konchwalla, Mohammed Sobhy Badr Sobei, Amal Hassan Hassan Ismail

**Affiliations:** 1 College of Medicine, Gulf Medical University, Ajman, United Arab Emirates; 2 Department of General Surgery, Thumbay Hospital, Ajman, United Arab Emirates; 3 Department of Obstetrics and Gynecology, Thumbay Hospital, Ajman, United Arab Emirates

**Keywords:** Torsion, Fallopian Tube, Adnexal Torsion

## Abstract

**Objective:** Isolated salpingeal torsion (IST) is a rare cause of acute abdomen in women of the reproductive age group. The lack of pathognomonic clinical, laboratory or radiographic findings makes early diagnosis a challenge. We describe a case of IST in a 13-year-old who suffered from a repeat torsion following initial conservative management.

**Case Report:** A 13-year-old girl presented with acute right lower quadrant abdominal pain of insidious onset. Her past medical history was non-contributory and her initial workup was unremarkable. Exploratory laparoscopy subsequently revealed isolated torsion of the right fallopian tube which was detorted. She presented 10 months later with similar complaints and further workup demonstrated right adnexal torsion which was confirmed by laparoscopy; salpingo-oophorectomy was necessary due to gangrenous necrosis.

**Conclusion:** Conservative management of fallopian tube torsion confers the advantage of fertility preservation but the risk of repeat torsion remains clinically significant.

## Introduction

Isolated salpingeal torsion (IST) is rare gynecological entity with an estimated incidence of 1:1.5 million that affects adolescent girls and women of the reproductive age group (1-7). The etiology of IST remains undetermined but certain intrinsic (congenital anomalies of the fallopian tube, hydrosalpinx/hematosalpinx, tubal neoplasms, abnormal tubal motility or spasms) and extrinsic (tubal or ovarian mass, adhesions, pregnancy, pelvic trauma or congestion) risk factors predispose to fallopian tube torsion. In children, isolated salpingeal torsion is typically associated with intrinsic risk factors (6). 

Intrinsic or extrinsic compression of the fallopian tube impairs venous and lymphatic flow causing congestion and edema; this increases the weight of the fimbrial end of the tube and subsequently, precipitates torsion (7). We present a case of fallopian tube torsion with repeat adnexal torsion after initial conservative management.

## Case report

A 13-year-old girl presented to the emergency department with severe right lower quadrant abdominal pain of insidious onset that started three days prior. The pain was non-radiating, crampy with no aggravating or relieving factors. She also reported fever, nausea, vomiting, dysuria and hematuria. Past health history, medication history, family history and sexual history were unremarkable. She attained menarche 6 months prior with irregular cycles. Initial examination findings were as follows: temperature-36.7 °C, blood pressure- 90/60 mmHg, respiratory rate-20/min and heart rate-86 beats/min; her abdomen was soft with right iliac fossa and suprapubic tenderness. There were no peritoneal signs. Her laboratory work-up revealed a white blood cell count of 12.7 × 10^3^/μl and 8-10/HPF white cells on urinalysis; urine pregnancy test was negative. Other laboratory investigations were unremarkable. Abdominal ultrasound revealed a large cyst (4.8 × 4 cm) in the right adnexal region. The uterus and left adnexa appeared normal. A computerized tomography (CT) scan confirmed the presence of a thinly walled, homogenous, right adnexal cyst (4.6 × 5cm) with mild free pelvic fluid; there was no evidence of acute appendicitis.

**Figure 1:**
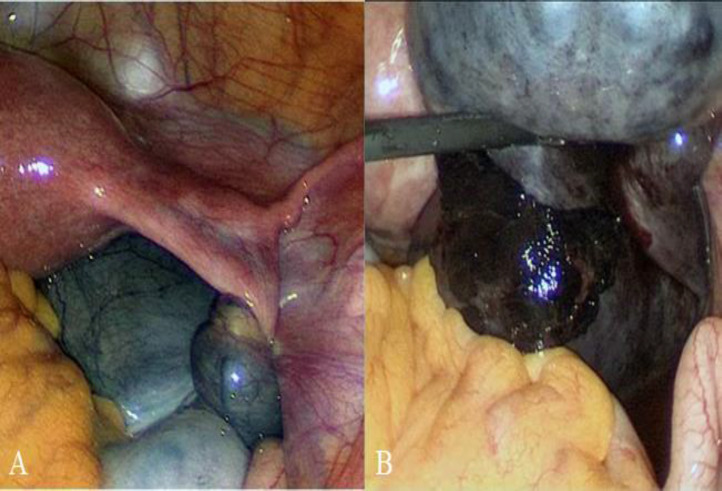
Laparoscopic image showing isolated torsion of the right fallopian tube with hydrosalpinx (A) and gangrenous right adnexal torsion (B) in a 13-year-old girl.

The patient was initially managed with intravenous fluids and analgesia. However, her pain progressively worsened and a decision was made to proceed with a diagnostic laparoscopy. Laparoscopy showed a dilated, torted and edematous right fallopian tube with normal looking right ovary, uterus and left adnexa (Figure 1). 

The fallopian tube was released (detorted), hydrosalpinx drained and blood flow was restored. She was discharged 72 hours post-operatively with no intraoperative or postoperative complications. Cytology report of hydrosalpinx fluid was negative for malignant cells.

The patient presented with similar symptoms 10 months later. Ultrasound evaluation revealed a right ovarian cyst (5.4 × 4.4 cm) with decreased vascular supply. Repeat torsion was suspected and diagnostic laparoscopy was performed immediately; it showed a gangrenous right adnexal torsion and an inflamed appendix with severe adhesions. Therefore, salpingo-oophorectomy and appendectomy were performed without complications and she was discharged after 48 hours. Histopathology subsequently confirmed a necrotic fallopian tube with no signs of malignancy and a benign serous cystadenoma of the ovary.

## Discussion

Although a number of risk factors have been associated with IST (1, 4-7), the etiopathogenesis is not always clear. Pediatric cases of IST are typically associated with intrinsic risk factors but it is uncertain whether the hydrosalpinx and unusually long fallopian tube we found during initial laparoscopy in our patient were the cause or consequence of tubal torsion. Notwithstanding, her subsequent adnexal torsion most likely resulted from the ovarian cyst (serous cystadenoma) coupled with a fallopian tube at risk of torsion. 

IST most commonly presents with convulsive pain that radiates toward the ipsilateral thigh and worsens over time. The right fallopian tube is more commonly affected possibly because the left fallopian tube is secured in place by the sigmoid colon and the right side is usually, more prone to clinical evaluation for suspicion of appendicitis (5, 6). Non-specific symptoms like nausea, vomiting, anorexia, fever, urinary symptoms and vaginal bleeding may also be present. Initial laboratory studies may be normal but mild leucocytosis and raised inflammatory markers are possible findings (4, 6). Pre-operative diagnosis remains a challenge because there are no pathognomonic features (1, 4, 5, 7). 

Ultrasound findings are highly variable as IST can appear as a complex adnexal mass, tubal wall thickening or dilation (1, 4-6). The uterus tends to be deviated towards the affected side. A swirling appearance (‘whirlpool sign’) is relatively specific in the right clinical scenario (5). Decreased or absent blood flow on colour Doppler is highly suggestive but not sensitive because of the dual adnexal blood supply (4-6). Magnetic resonance imaging and computed tomography scan may show tubal coiling (‘coiling sign’). Imaging allows early intervention depending on specific findings and can also rule out other surgical causes of acute abdomen (1, 2, 4-6). Laparoscopy remains the gold standard for definitive diagnosis (1, 4, 5, 7).

The management of IST is contentious because of its potential impact on future fertility. Conservative management with de-torsion (+/- drainage of hydrosaplinx) is increasingly performed in place of the more radical salpingectomy. Although fertility preservation is a justifiable therapeutic endpoint of IST treatment, the long-term outcomes of conservative management remains undermined (5) while the potential morbidity and mortality of repeat torsion persists as revealed in our case. Salpingopexy has been proposed to decrease the risk of repeat torsion but there is limited evidence on its benefits or potential impact on residual fallopian tube and ovarian function. Clinical trials and large data repositories are necessary to characterize the outcomes of conservative vs. radical management of IST. Furthermore, risk stratification based on the predictors of repeat torsion is a more objective criterion for treatment selection on a case by case basis. Until such evidence becomes available, a transdisciplinary approach that includes pediatric surgeons and gynecologists is imperative to optimize the outcomes of pediatric IST cases.

## Conclusion

There are no pathognomonic clinical or imaging findings of isolated fallopian tube torsion which makes timely diagnosis and management a challenge. Depending on the severity of torsion and tissue integrity, management of IST can significantly impact future fertility. Transdisciplinary approach with pediatric surgery and pediatric gynecology is necessary to improve the outcomes of pediatric IST cases. 

## References

[1] Krissi H, Shalev J, Bar-Hava I, Langer R, Herman A, Kaplan B (2001). Fallopian tube torsion. J Am Board Fam Pract..

[2] Harmon JC, Binkovitz LA, Binkovitz LE (2008). Isolated fallopian tube torsion: sonographic and CT features. Pediatr Radiol.

[3] Lo GC, Kadoch MA, Simpson W Jr (2016). Isolated fallopian tube torsion: two case reports of a rare entity. Clin Imaging.

[4] Casey RK, Damle LF, Gomez-Lobo V (2013). Isolated fallopian tube torsion in pediatric and adolescent females: a retrospective review of 15 cases at a single institution. J Pediatr Adolesc Gynecol.

[5] Bertozzi M, Magrini E, Riccioni S, Giovenali P, Appignani A (2017). Isolated fallopian tube torsion with hydrosalpinx: Review of a debated management in a pediatric population. J Pediatr Surg.

[6] Orazi C, Inserra A, Lucchetti MC, Schingo PM (2006). Isolated tubal torsion: a rare cause of pelvic pain at menarche. Sonographic and MR findings. Pediatr Radiol.

[7] van der Zanden M, Nap A, van Kints M (2011). Isolated torsion of the fallopian tube: a case report and review of the literature. Eur J Pediatr.

